# Debris Removal from Mandibular Molars Using Different Irrigation Volumes and Ultrasonic Tips: Micro-CT Study

**DOI:** 10.4317/jced.62892

**Published:** 2025-08-01

**Authors:** Alinne Patiery Pacífico Oliveira Feitosa, Arthur Costa Lemos, Suyane Maria Luna-Cruz, Ana Grasiela Limoeiro, Murilo Priori Alcalde, Rodrigo Ricci Vivan, Marco Antônio Hungaro Duarte, Bruno Carvalho Vasconcelos

**Affiliations:** 1 Post-graduate Program in Dentistry, Faculty of Pharmacy, Dentistry and Nursing, Federal University of Ceará, Fortaleza, CE, Brazil; 2 Department of Dentistry, Endodontics and Dental Materials, Bauru Dental School, University of São Paulo, Bauru, SP, Brazil

## Abstract

**Background:**

This study compared the impact of varying irrigating solution volumes and two ultrasonic tips on removing hard tissue debris (HTD) during passive ultrasonic irrigation (PUI) in mesial roots of mandibular molars.

**Material and Methods:**

Sixty roots were instrumented using WaveOne Gold 35/.06 divided into 5 groups (*n*=12): Irrisonic 6 mL (IR6), Irrisonic 30 mL (IR30), Irrisonic Power 6 mL (IRP6), Irrisonic Power 30 mL (IRP30), and a control group with conventional irrigation. Microcomputed Tomography was used to measure HTD removal. Statistical analysis used ANOVA, Tukey, and t-Student tests (α = 5%).

**Results:**

Significant differences were observed in HTD removal between the IR30, IRP6, IRP30 groups, and the control group (*P* < 0.0001), while no statistical significance was found between IR6 and the control group (*P* > 0.05). A statistical difference was observed between IR30 and IR6 (*P* < 0.05). IR30 achieved the highest debris removal rate (87.63%), while IR6 showed the lowest (80.16%). Significant differences were observed between experimental and control groups (*P* < 0.05), except for IR6 (*P* > 0.05).

**Conclusions:**

A larger volume of irrigating solution (30 mL compared to 6 mL) during the PUI protocol resulted in greater removal of HTD. Additionally, the Irrisonic Power tip did not significantly enhance HTD removal.

** Key words:**Endodontics, Passive Ultrasonic Irrigation, Root Canal Irrigant, Ultrasonic tip, X-ray microtomography.

## Introduction

The presence of hard-tissue debris (HTD) in root canal during chemo-mechanical preparation can negative impact root canal desinfection, particularly in the apical third and areas with anatomical irregularities [1,2]. Isthmus morphology can complicate root canal debridement due to its narrow extensions between canals [3], potentially harboring tissue remnants, microorganisms, and HTD [3], which can lead to treatment failure [4].

Effective irrigation is crucial for cleaning complex anatomical areas, warranting special attention [1,2,5]. Sodium hypochlorite (NaOCl) is the most frequently mentioned irrigating solution in the literature, mainly because of its antimicrobial properties [6,7] and its ability to dissolve organic matter through direct contact [8]. The contact time and volume during irrigation directly influence its efficacy, especially in hard-to-reach areas [7,9]. However, a standardized protocol for irrigation, including volume, application time, and activation methods, is lacking in the literature, which could enhance endodontic treatment predictability [10]. PUI protocols that incorporate different volumes of irrigant and various ultrasonic tips can address this need.

Passive ultrasonic irrigation (PUI) was introduced to improve the performance of irrigating solutions compared to conventional irrigation. Previous studies have indicated a significant reduction in debris with PUI as opposed to conventional needle irrigation [5,11]. The PUI technique requires the use of ultrasonic tips (UTs), which are typically thin and smooth, and used in a passive push-pull circumferential motion to avoid contact with the root canal walls [12].

Irrissonic (Helse Ultrasonics, Santa Rosa do Viterbo, Brazil) is a stainless-steel rod-shaped UT with a tapered tip matching that of a size 20.01 instrument [5,13]. The manufacturer’s recommended power setting is 10% to avoid early fractures due to its caliber [13]. Despite its good results [5,14], proposed design changes, particularly in length, point, and bending angle, have led to the development of the Irrisonic Power (Helse Ultrasonics). According to the manufacturer, these modifications aim to enhance the instrument’s resistance, potentially improving the cleaning of the root canal system (RCS) [13].

Recognizing the importance of effective cleaning of the root canal system is essential for the success of endodontic treatment, in this sense, the question arises as to whether the modified ultrasonic tip or the solution volume affects the ability to remove HTD from mandibular molar canals and isthmuses. The present study aimed to evaluate the removal of HTD from mesial canals of mandibular molars with isthmus using PUI protocols with different volumes of irrigant and different UTs. The null hypothesis is that neither a larger volume of irrigating solution nor the use of a modified UT will increase HTD removal.

## Material and Methods

- Specimen selection and preparation

This study received approval from the Local Research Ethics Committee (approval number 4.597.242). The manuscript of this laboratory study has been written according to Preferred Reporting Items for Laboratory studies in Endodontology (PRILE) 2021 guidelines [15].

After sample size calculation, 60 human mandibular molars, extracted for reasons unrelated to this research, were selected based on the following criteria: apparent straight roots (curvature less than 5°) according to Schneider [16] measuring between 18 and 21 mm, complete rhizogenesis and apicigenesis, and absence of calcifications. Teeth with previous endodontic treatment, root fractures, or apical foramina larger than 200 µm were excluded.

The crowns were sectioned at the cementoenamel junction, and the mesial roots were separated using a Carborundum wheel (Dentorium, New York, USA) attached to a handpiece (Dabi Atlante, Ribeirão Preto, Brazil). The roots were standardized to 15 mm in length, confirmed using a digital caliper with a precision of ± 0.001 mm (FNCL, Worker Gage, Esteio, Brazil).

Based on a previous study [17], the roots were scanned using microcomputed tomography (micro-CT) (SkyScan1174; SkyScan, Aartselaar, Belgium) with a 19 µm voxel size, 50 kV, 800 mA, 0.8º rotation, and 1024 x 1304 resolution. This procedure allowed the identification of Type II or V isthmuses according to the classification by Hsu & Kim [18]; roots that did not meet this pattern were replaced. Foraminal patency and canal length were verified using #15 K-type manual files (Dentsply-Sirona, Ballaigues, Switzerland). The root apices were sealed with utility wax and adapted to a metallic support for stabilization. Canal preparation was standardized, extending to 1 mm short of the real canal length (RCL). Before instrumentation, the specimens were irrigated with 2 mL of 2.5% NaOCl (Asfer Industria Química Ltda, São Caetano do Sul, Brazil). WaveOne Gold Medium instruments (#35/.06; Dentsply-Sirona) driven by an electric motor (VDW Silver; VDW GmbH, Munich, Germany) were used in the “WAVEONE ALL” setting. Instrumentation was performed through cycles of 3 pecks using slow in-and-out movements with smooth brushing against the mesial surface. After each cycle, the canals were irrigated with 2 mL of 2.5% NaOCl. At each pecking sequence, the instrument was cleaned with gauze and the RCL was recapitulated using a manual #20 K-file. All irrigation steps, regardless of the solution used, were performed using a disposable luer syringe (BD, Juiz de Fora, Brazil) adapted to a specific irrigation needle (NaviTip 29G; Ultradent, South Jordan, UTAH, USA) with a file stop adjusted to 2 mm short of the working length.

After the chemo-mechanical preparation, new micro-CT scans were performed to confirm the presence of HTD in the canals and isthmuses. The volume of the HTD was determined before the random division of the specimens among the four experimental and the control groups. To ensure standardization, the HTD volume present in groups was analyzed using ANOVA test, which showed no statistical differences among them (*P* > 0.05).

- Final irrigation protocols

The Irrisonic and Irrisonic Power tips were coupled to a piezoelectric ultrasonic device (Newtron P5XS B.LED; Acteon Satelec, Merignac, France). They were activated with 10% power and inserted up to 2 mm short of the working length, using in-and-out movements in the buccolingual direction, without touching the canal walls. The following 2.5% NaOCl solution agitation protocols were used:

IR6 - Irrisonic tip with a total of 6 mL of irrigating solution, distributed in three cycles of 20 seconds with 2 mL each.

IR30 - Irrisonic tip with a total of 30 mL of irrigating solution, distributed in three cycles of 20 seconds with 10 mL each.

IRP6 - Irrisonic Power tip with a total of 6 mL of irrigating solution, distributed in three cycles of 20 seconds with 2 mL each.

IRP30 - Irrisonic Power tip with a total of 30 mL of irrigating solution, distributed in three cycles of 20 seconds with 10 mL each.

Control group - Using a disposable syringe and needle, 6 mL of irrigating solution was distributed in three cycles of 20 seconds with 2 mL each.

After the NaOCl irrigation cycles, three agitation cycles with 17% ethylenediaminetetraacetic acid (EDTA) were performed, totaling 6 mL; each group used the previously allocated tip. The canals were then flushed with 5 mL of saline solution (Asfer Indústria Química Ltda) and dried with absorbent paper. It is notable that the total irrigation volume per canal, from preparation to final irrigation, totaled 25 mL in the IR6/IRP6 groups and 49 mL in the IR30/IRP30 groups. Upon completion of the preparation and final irrigation protocols, new micro-CT scans were performed as previously described.

- Debris analysis

The images from the micro-CT scans were reconstructed using NRecon 1.6.9.16 software (Bruker micro-CT) and aligned with the 3D registration function of the DataViewer software (v.1.5.1; Bruker micro-CT). The images were then processed with the CTAn software (v.1.14.4; Bruker microCT) for calculating quantitative parameters and the constructing of 3D visual models. The amount of HTD was calculated as reported previously [19]. The volume of interest for each specimen extended from the furcation region to the apex of the mesial root, defined by integrating the regions of interest in all cross-sections of the analyzed tooth.

The grayscale range required to recognize dentin before and after instrumentation and after final irrigation was determined using a density histogram with a global threshold method. Comparisons between the original and segmented scans ensured segmentation accuracy. The volume of the remaining HTD after the final irrigation protocols was determined by superimposition with the original scan, comparing the post-instrumentation image with the image after the final irrigation protocols. The percentage reduction of debris (Rd) was calculated using the formula: %Rd = ((A - B)/A) * 100, where A is the volume of debris before final irrigation and B is the volume after final irrigation.

- Statistical Analysis 

The normality of the percentage values obtained for HTD removal was tested using the Kolmogorov-Smirnov test. ANOVA and Tukey tests were applied to compare the experimental and control groups. The isolated factors, the volume of solution, and the type of UT were compared using the t-Student test. A 5% significance level was considered for all analyses.

## Results

**Figure 1:**
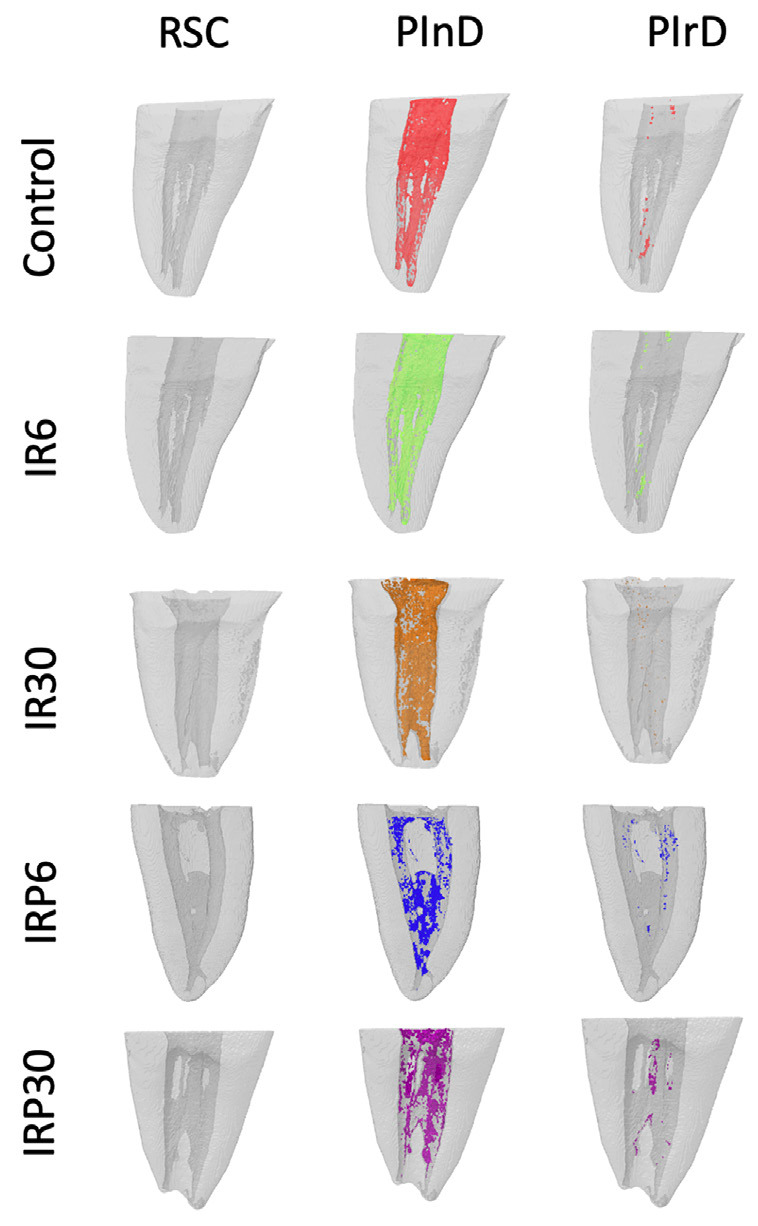
RCS – Root Canal System. PInD – Post-instrumentation Debris. PIrD – Post-irrigation Debris. Control – Conventional Irrigation. IR6 – Irrisonic + 6 mL irrigating solution. IR30 – Irrisonic + 30 mL irrigating solution. IRP6 – Irrisonic Power + 6 mL irrigating solution. IRP30 – Irrisonic Power + 30 mL irrigating solution. Images generated using CTVol 2.2.3.0 softwares.

Figure 1 shows illustrative images of the 3D reconstructions of the evaluated HTD removal protocols. The results comparing the performance of the protocols in removing HTD from mandibular molar canals and isthmuses are described in  Table 1. Significant differences were observed in HTD removal between the IR30, IRP6, IRP30 groups, and the control group (*P* < 0.0001), while no statistical significance was found between IR6 and the control group (*P* > 0.05). A statistical difference was observed between IR30 and IR6 (*P* < 0.05).

Individual analysis of the variables revealed a statistical difference in HTD removal between the smallest (6 mL) and the largest volume (30 mL) (*P* < 0.0001); however, no statistical significance was observed between the different UTs (*P* > 0.05).

## Discussion

The present study aimed to evaluate the influence of different volumes of irrigating solution and UTs on the removal of HTD from root canals and isthmuses of human mandibular molars. To date, there has been no report in the literature evaluating similar volume variations or changes in the design of the ultrasonic agitation tip under controlled conditions. Based on the results of this study, the null hypothesis was partially rejected since the use of a greater volume of irrigating solution proved to be more effective in removing debris.

We chose to use mesial roots of mandibular molars because of the higher frequency of isthmuses in these teeth, as reported in the literature [5]. In a review of 15 studies [20], it was found that isthmus communications in the mesial roots of mandibular molars occurred in 54.8% of a sample of 1615 teeth. This anatomical variation is considered a challenge for effective cleaning of the root canal system in clinical practice [5,17], as it can impede disinfection procedures, preventing the flow of irrigation and diminishing the antibacterial effects of the irrigating solution [21]. The volume of irrigating solution used in the control group was based on the literature standards to serve as comparison for cleanliness achieved by PUI protocols with lower irrigation volumes. The validity of increasing the volume of solution was thus verified [13]. Additionally, preliminary micro-CT scans enabled group division by type of isthmus, classified as Types II [18]. By standardizing the isthmuses, potentially anatomical biases that could interfere with the study results were reduced [5].

Greater removal of debris was observed when PUI was used compared to the control group, except in the IR6 group. Previous studies had already noted this superiority [1,5,17] however, non-significant results were also reported in the literature [22]. Despite the satisfactory HTD removal results observed, the similarity between the control and the IR6 groups was unexpected. A possible explanation is the greater effectiveness of syringe irrigation in cavity preparations with a larger enlargement of the apical third [23], as in the present study where the enlargement was caried out up to size #35/.06.

It is noteworthy that, although significant debris removal from the root canals and isthmuses was observed, no irrigation technique achieves complete removal of HTD [5,17,24] which was also observed in this study. Such variation in results is discussed in a systematic review [25], which highlights the lack of standardization among studies that use ultrasonic activation and variability in analysis methods, factors that may influence the result.

Following the NaOCl irrigation cycles, three agitation cycles with 17% EDTA were conducted, totaling 6 mL, with each group using the designated tip assigned beforehand. Studies that utilized the same irrigating solutions, typically NaOCl combined with EDTA, but employed varying activation protocols, have also shown the impact of these protocols on the removal of accumulated hard tissue debris from root canals [26,27].

Regarding the effect of the volume of irrigating solution used (6 mL and 30 mL), the results indicated a significant increase in debris removal with the greater volume. These findings corroborate previous studies where a larger volume significantly removed more biofilm from isthmus-like structures [7,9]. It is understood that the volume of the irrigant, influenced by the irrigation-aspiration flow, directly impacts its action, especially in hard-to-reach regions such as isthmuses. The GentleWave System, which uses a large volume of irrigant, was effective in removing biofilm from the mesial roots of mandibular molars both before and after minimal instrumentation [28]. However, a study by Van der Sluis *et al*. [29] showed no significant difference concerning volume variation (6, 12, and 50 mL); in the study, the authors also varied the method of solution insertion, using continuous flow or with differing irrigation time intervals (30 s or 60 s). It is suggested that the amplitude of the preparation performed may justify the divergence in results observed (#20.01 vs. #35.06) where more space available allowed better cleaning with PUI [23].

Regarding the use of Irrisonic and Irrisonic Power UTs, no significant difference was observed. According to Dos Reis *et al*. [30], the difference between these two tips lies in their manufacturing process, rendering the Irrisonic Power tip more resistant. The geometry, length, and tip shape of the insert can affect how the ultrasonic energy is transmitted through the irrigant, generating effective acoustic streaming that enhances the movement of the fluid within the canal. This streaming helps dislodge debris and biofilm. Additionally, turbulence created by the insert’s design can improve contact between the irrigant and the root canal surfaces, further enhancing debris removal and improving the efficacy of the irrigation process. Future studies are suggested to verify whether this new tip can withstand greater power settings while also improving cleaning capacity.

The clinical take-home message from this study is that larger volumes of irrigating solution, even after chemo-mechanical preparation, result in more effective root canal system cleaning, potentially increasing the success rate of the endodontic treatments. This finding reinforces the concept that the canals should be adequately prepared before cleaning during endodontic treatment. However, further studies using even larger volumes of irrigating solution or higher power ultrasonic agitation are needed to verify whether these variations could further enhance tissue debris removal.

Based on the experimental conditions of this study, it can be concluded that the use of a larger volume of irrigating solution (30 mL vs. 6 mL) during PUI protocol yielded a greater HTD removal. Furthermore, the Irrisonic Power tip did not significantly increased HTD removal.

## Figures and Tables

**Table 1:** Table Percentages (mean and standard deviation) of hard tissue debris reduction provided by final irrigation protocols.

Volume	Irrisonic		Irrisonic Power		p-Valueb
	Mean	sd	Mean	sd	
6 mL	80.16	6.17	85.82	5.46	0.0833
30 mL	87.63	4.32	86.95	4.69	0.9978
p-Valuea	0.0096		0.9853		
Control	74.23	5.76			
p-Valuec	0.0623		<0.0001		
p-Valued	<0.0001		<0.0001		

a ANOVA/Tukey tests for the same tip (6 mL vs. 30 mL); b ANOVA/Tukey tests for the same volume (Irrisonic vs. Irrisonic Power); c ANOVA/Tukey tests for different tips with 6 mL vs. Control; d ANOVA/Tukey tests for different tips with 30 mL vs. Control.

## Data Availability

The datasets used and/or analyzed during the current study are available from the corresponding author.
